# Assessment of Knowledge of Critical Cardiovascular Risk Indicators among College Students: Does Stage of Education Matter?

**DOI:** 10.3390/ijerph14030250

**Published:** 2017-03-02

**Authors:** Daniel F. Sarpong, India Y. Curry, Melinda Williams

**Affiliations:** 1 Center for Minority Health and Health Disparities for Research and Education, Xavier University of Louisiana, 1 Drexel Drive, New Orleans, LA 70125, USA; 2 College of Nursing, University of South Alabama, 5721 USA Drive N. Room 3068, Mobile, AL 36688-0002, USA; mscurry14@yahoo.com; 3 School of Health Sciences, Jackson State University, 305 W. Woodrow Wilson Ave, Jackson, MS 39213, USA; Melinda.n.williams@students.jsums.edu

**Keywords:** cardiovascular disease, Know Your Numbers, young adults, college students, prevention

## Abstract

The health risk of college students in the United States (US) is on the rise, with a significant increase in the prevalence of cardiovascular risk factors. Cardiovascular disease is the leading cause of death in the US, costing approximately $475.3 billion yearly. The goals of this “Know Your Numbers” study were to: (1) estimate the awareness of college students of their critical health numbers (CHN); and (2) compare a college of pharmacy entry class (IP1) with second semester non-commuter freshman college students (FCS) in knowing their numbers. A cross-sectional 15-item pre-test survey was conducted among a convenience sample of IP1 and FCS. All statistical tests were performed at α = 0.05. Awareness of their: cholesterol (7%), blood pressure (BP) (35%), glucose (8%), and body mass index (BMI) (42%) were low. The IP1, compared to FCS, were more knowledgeable of: (1) their BP (46% vs. 28%, *p* = 0.01); (2) BP normal range (74% vs. 63%, *p* = 0.02); and (3) BMI normal range (39% vs. 23%, *p* = 0.04). The IP1s maintained a healthier diet than the FCS (64% vs. 36%, *p* < 0.0001). Awareness of knowing CHN was very low. Knowledge of one’s CHN was significantly associated with knowledge of normal reference values for BP, glucose, and BMI.

## 1. Introduction

The level of awareness of cardiovascular disease (CVD) among college-age students is limited and understudied [1]. Also understudied is the gender- and ethnic-differences in CVD among this population [1]. American college students faced with new-found freedoms and responsibilities, such as eating whatever they want and being physically active, have the challenge of making the right decisions for their health [2]. It is easy to make poor choices and engage in risky behaviors when confronted with making the right choices for the first time in their lives [3]. However, this situation provides an excellent opportunity to present educational material on healthy lifestyle choices. Healthy behaviors formed during young adulthood can have long-lasting effects on health later in life [3].

In the United States (US), on an annual basis, nearly 630,000 individuals die from some form of heart disease, and over 4.2 million individuals are hospitalized. Cardiovascular disease (CVD) is the leading cause of death in both men and women, and it is more costly than cancer, with an estimated cost of $475.3 billion yearly [4]. Furthermore, the most important way to reduce CVD incidence is through preventative methods, which include modifying risk factors and education [4]. The age of onset of CVD occurs very early in life and influenced by many factors over time. These factors are (a) lifestyle choices and behaviors, (b) environments where persons live, (c) and modifiable risk factors such as high blood pressure, smoking, high cholesterol, obesity, type 2 diabetes, and physical inactivity [5]. Although CVD has been an issue in the US for many years, awareness of the disease remains a major concern. From 2009 to 2010, 81.9% of adults were aware that they had hypertension (HTN); however, awareness in young adults (18–39) was lower than awareness in adults 40 or older. Over a 10-year span from 1998 to 2008, awareness, treatment, and control of hypertension for adults with diabetes (DM) increased, but there was no evidence of improvement for adults aged 20 to 44 years. In 2005–2006, 40% of adults over 20 years of age did not know they had DM [5].

There is compelling evidence in the literature that the target population for this study has a significantly higher risk for CVD than they are aware of, and early intervention should mitigate future disparities in CVD and other co-morbid conditions. According to a study by Winham and Jones (2011), of 172 African American (AA) young adults, 40% had two or more risk factors associated with CVD [6]. Keating et al. (2005) estimated that 50% of college students did not engage in physical activity [7]. Collins and colleagues (2004) [8] found that college students overall had relatively low levels of knowledge about heart disease and its risk factors compared to other health issues, such as sexually transmitted diseases and psychological disorders. Programs designed to increase knowledge about CVD have been implemented all over the United States. Specifically, the ‘Know Your Numbers’ (KYN) program, designed to encourage people to know their cholesterol, blood sugar, blood pressure and body mass index numbers, will promote self-assessment of health and personal responsibility in avoiding CVD complications in the future.

These KYN campaigns are in accordance with the recommendations and advocacy by the American Heart Association (AHA) and the World Heart Federation (WHF) of people being aware of their numbers for cardiovascular health [9,10]. Because systolic and diastolic blood pressures, blood glucose (BG), BMI, and total cholesterol (TC) are all biomarkers for hypertension, diabetes mellitus, obesity, and dyslipidemia, respectively, AHA advocates knowledge of the values of these biomarkers will allow a person and his/her provider to determine his/her risk for developing CVD [9,10]. Knowledge and tracking of one’s health numbers over time empowers and increases understanding of how to improve one’s health [9]. The goals of this “Know Your Numbers” study were to: (1) estimate the level of awareness of college students of their numbers (critical health numbers); and (2) compare a college of pharmacy entry class with second semester college freshmen in terms of knowing their numbers. The primary hypothesis was that people who are going into a healthcare profession are more aware of their numbers when compared to incoming college freshmen. Additionally, we hypothesize that there is an association between knowledge of cholesterol, blood sugar, blood pressure and body mass index and knowledge of normal-reference values for these biomarkers.

## 2. Methods

### 2.1. Study Participants

The project participants were first-time college freshman students, both males and females (18–20 year olds) and an incoming Professional Year 1 (IP1) Pharmacy students at a Historically Black College and University (HBCU) located in Louisiana. A total of 133 second-semester freshman and non-commuter students and 90 IP1 students were recruited. Freshman students were not enrolled if they had not completed at least one semester of college; because they would not have had adequate time outside of their home to assess the impact of being independent from parents/guardians on their health and lifestyle. All project participants consented before enrollment.

### 2.2. Data Collection

Institutional Review Board (IRB) approvals were obtained from the University of South Alabama and Xavier University of Louisiana. To maintain confidentiality, all consented project participants used an algorithm described on the KYN pre-test survey for generating their unique Participant Identification Number (PID). The PID consisted of their initials and the last four digits of their student ID. All survey data collected were anonymous. Completion of the baseline survey lasted approximately 5–7 min and was administered either in a group setting in selected sections of the Freshman Seminar II courses or to individual students at the residency halls. Using a listserv, students in the residency halls were recruited via email. Students were informed of the study and were invited to contact their residency hall assistants to consent and complete the survey on set dates and times. Two residency hall assistants (RHAs) trained as research assistants for the project, ensured that eligible freshmen consented before participating in the self-administrated survey. Because data collection started after consenting to participate in the study, we were not able to assess response rate or characteristics of eligible freshmen who chose not to participate in the study. For the IP1 students, the survey was administered in a summer enrichment program, which was the summer before the start of their Professional Year 1. The project investigator and research mentor were the data collectors for the IP1 data. There was no cost to the students in participating in this project, and the students were informed that they could discontinue the program at any time. Data was initially captured in Microsoft EXCEL and then retrieved and managed in SAS 9.4. (SAS Institute, Cary, NC, USA).

### 2.3. Study Variables

Study measures included demographics, knowledge of participant’s health-related numbers (e.g., glucose, cholesterol, blood pressure and body mass index), and health behaviors (e.g., physical activity and diet) and knowledge of the normal values of health-related numbers of healthy adults. The primary independent variable was student classification (second semester freshman versus IP1 students). The covariates were demographic factors.

### 2.4. Statistical Analysis

Descriptive statistics were used to describe characteristics of the study sample. Two-sample *t*-tests were used to assess differences in subgroups defined by academic classification (freshmen and incoming P1) for continuous measures. Chi-square tests were used to test for association between academic classification and categorical measures. Simple and multiple logistic regression models were used to determine unadjusted and adjusted associations between knowledge of one’s numbers and knowledge of normal reference values of the critical health numbers. All statistical tests were performed at a significance level of 0.05 using SAS 9.4 (SAS Institute, Cary, NC, USA).

## 3. Results and Discussion

### 3.1. Results

There were differences in gender, age and race/ethnicity distributions between the two student groups. The IP1s compared to freshmen had fewer females (70% vs. 87%), fewer AAs (38% vs. 92%), and were older (percent older than 24 years; 81 vs. 0). Although the overall percentage of participants’ knowledge of their numbers were low; ranging from 7% to 42%, there were differences between IP1s and freshmen in terms of knowledge of total cholesterol (10% vs. 5%); BP (46% vs. 28%); and percentage of participants with all four health indicators in normal range (31% vs. 17%). Similarly, a greater percentage of the IP1s compared to freshmen was aware of their BP (74% vs. 63%) and BMI (39% vs. 23%). On average the IP1s engaged in physical activity a little more than freshmen (2.7 ± 1.7 vs. 2.3 ± 2.1 days per week); and a greater percentage of the IP1s indicated that they maintained healthy diets compared to freshmen (64% vs. 36%). Table 1 provides a summary of the characteristics of the study sample.

Table 2 provides a comparative analysis of key outcome measures of knowing one’s numbers. The IP1s compared to the freshmen, were more knowledgeable of: (1) their BP (46% vs. 28%, *p* = 0.01); (2) the normal range for BP (74% vs. 63%, *p* = 0.02); and (3) normal range for BMI (39% vs. 23%, *p* = 0.04). The IP1’s maintained a healthy diet at a lower percentage than the freshmen (36% vs. 64%, *p* < 0.001).

Table 3 provides a summary of the associations between the students’ knowledge of their health numbers and their knowledge of the reference ranges of the health numbers of a healthy adult. Based on the fully adjusted model (Model II), the following significant associations were realized for BP (OR = 6.07; 95% CI: 2.69 to 13.66); blood glucose (OR = 3.1; 95% CI: 1.1 to 8.3); and BMI (OR = 4.2; 95% CI: 2.2 to 7.8). Study group (IP1s vs. freshmen) was positively associated with knowledge of reference range of BMI in model I, however, the association was fully attenuated when the covariates were accounted for in the fully adjusted model. In the models, the association between knowledge of one’s BP and knowledge of reference range of BP for a healthy adult, age was the only significant covariate (OR = 2.3; 95% CI: 1.0 to 5.1).

### 3.2. Discussion

Overall, the level of awareness of the target population of their health-related numbers was extremely low for all four indicators (total cholesterol, blood glucose, blood pressure and body mass index); particularly for total cholesterol and blood glucose. It is also alarming that less than one in every four of the students knew if their four health indicators were within normal range. This result supports the findings of Collins and colleagues (2004) [8] who found college students had both a low risk perception and low knowledge level about heart disease compared to other health issues [8]. The female and minority students did not identify themselves at high risk for heart disease [8]. Similar to the findings of Collins et al. (2004) [8], Becker and colleagues (2008) found that college students were unaware of metabolic syndrome or CVD risk factors. More disturbing is that some college students hold false beliefs about CVD complications [11]. Part of the explanation could be that students at this stage of their lives feel that heart disease and related risk factors are more in the distant future. Given the age, gender and race/ethnicity distributions of the two study groups, one might suspect that there are age, gender and racial/ethnic differences in the level of awareness of critical health numbers. However, only age was a significant covariate for BMI when these covariates were accounted for in the series of multiple logistic regression models whose results are summarized in Table 3.

Less than 25% of the students surveyed indicated that their values of all four of the CVD-related critical indicators were within normal range. This statistic raises concern that these young adults are at greater risk of developing CVD than perceived by them or society. This finding is supported by the results of a study by Holland et al. (2014) [2]. Holland and colleagues found that among African American college students, CVD risk factors such as elevated cholesterol, high blood pressure, family history of heart disease, overweight/obesity, physical inactivity, diet high in fat and sodium, and tobacco use mimic national trends [2]. Results from this study and that of Holland and colleagues underscore the fact that awareness and prevention of risk factors for CVD in African-Americans are critical even in the young adult population. This finding is troubling but supports results from a study by Spencer (2002) where traditional college students were screened for heart disease risk factors [12]. In that study, college students were found to be at risk for heart disease. Specifically, 29% of the participants had undesirable total cholesterol (TC) levels and up to 18% were above recommended levels for TC, high-density lipoprotein (HDL) cholesterol, and TC and HDL ratio. They were also at risk for high blood pressure (BP) since 15%–21% and 10%–11% had borderline BP levels and high systolic or diastolic BP levels, respectively. Additionally, 50% or more of the students had at least one parent diagnosed with high cholesterol or blood pressure [13].

Although the IP1s consistently had higher scores than the freshmen in terms of the four CVD risk factor indicators, only the knowledge of one’s blood pressure was significant (18 percentage point differential; *p* = 0.01). These findings might suggest that students pursuing professional careers might have advantage over college freshmen. Yahia et al. (2014) found that students majoring in the Health-Sciences scored 7 percentage points higher than non-Health-Science majors (*p*  <  0.001) as they had hypothesized [13]. However, in this study the IP1s were much older, had two or more years of college education and were more racial/ethnically diverse. Although, in both groups, less than 50% of students knew their BMI, a greater percentage (16% points; *p* = 0.04) of the IP1s knew their BMI versus the freshmen. In terms of knowing the reference normal values for the four indicators, normal blood pressure reference values were the only health indicators that more than 60% of students in both groups knew. The percentage of the correct response for the other three was low; ranging from 23% to 39%. It is not clear why knowledge of BP normal reference was higher than the other three health indicators. One possible explanation is the high prevalence of high BP in the nation. However, if this line of reasoning is correct, then one would have expected a greater percentage of the students to know the normal reference values for BMI, given the high rates of obesity in the nation.

When asked the basic question, “Do you maintain a healthy diet?” only 36% of the freshmen compared to 64% of the IP1s indicated yes. This finding is not shocking given that others have found similar results. DeBate (2001) found in his student sample that about 18% consumed 5 servings of fruits and vegetables, 6.9% consumed 6 grain products, and 53.1% consumed 2 servings of dairy products in the last 24 h [14]. Approximately 52% of the students in the study by Spencer (2002) consumed diets high in saturated fats, and greater than 30% of them did not consume adequate amounts of fiber [12]. The level of physical activity of the freshmen and the IP1s was much lower than what the Centers for Disease Control and Prevention (CDC) and American College of Sports Medicine recommend. The recommended goal is a minimum of 150 min of moderate physical activity each week or approximately 30 min of exercise at least five days per week. The two groups averaged 2–3 days (freshmen vs. IP1: 2.28 ± 2.14 vs. 2.66 ± 1.68 days; *p* = 0.1469) of physical activity. This is consistent with other studies [7,12,15,16,17,18,19].

In assessing the associations between knowing one’s critical health numbers and knowledge of the reference values for total cholesterol (TC), blood pressure (BP), blood glucose (BG) and body mass index (BMI) among healthy adults, we found that knowledge of one’s critical health numbers for BP, BG and BMI were significantly associated with the normal reference values of the respective health indicators based on fully adjusted logistic models. This was not the case for TC. However, for BP, the age of the student was also a significant predictor of knowledge of normal reference values for BP. Gender, education, race/ethnicity and study group (freshmen vs. IP1) were not significant covariates when they were adjusted for in the models. This is important because as much as we want young adults to know their numbers, they also need to know the normal reference values for the four CVD-related health indicators to determine if they are at risk or not. It is important to note that group membership was significant when examining knowledge of one’s BMI and its association to knowledge of normal reference values for BMI. However, the association was attenuated when gender, age, education and race/ethnicity were accounted for in the model. This suggests that the four demographic factors mediate the effect of group membership in its relationship to knowledge of normal reference values for BMI. This makes sense since the gender, age and race/ethnicity of freshmen and the IP1s are very different.

Although the study has strengths and adds significantly to the body of knowledge in an area of research understudied [1], it is important to discuss some potential limitations of the study. On the issue of potential selection bias, we could not say there was bias because we were unable to compare the characteristics of students who opted to versus those who did not participate in the study. However, the gender and racial/ethnic breakdown of the freshmen were consistent with the overall gender distribution of the undergraduate student population at the institution. Similarly, the gender and racial/ethnic distributions of the IP1 students were the same as past incoming first year pharmacy students in the college. Although we did not perform a power analysis prior to conducting the study, the sample size of 223 appears sufficient for our logistic regression analysis. A rule of thumb for determination of sample size for such analysis is a minimum of 10 observations per predictor variable in the model [20]. Since the maximum number of predictor variables was six (See Table 3), the required minimum sample size would be 60. One explanation for the wide 95% confidence intervals for the odds ratios reported in Table 3 is that the variability in the measures was large. Given that health disparities and health outcomes are significantly impacted by socioeconomic status [21]; there is a need to discuss its impact on the study findings. Hence, there might be concern that socioeconomic status (SES) of the two student groups might explain some of the findings. However, since SES data was not collected in this study it will be hard to comment of the role of SES. Nevertheless, one might infer that the SES of the two student groups might not differ much despite the large difference in the cost obtaining a pharmacy education versus a non-pharmacy education. The reason SES might not be a factor is that approximately 90% of students fund their pharmacy education with loans, thereby having an average debt of about $100,000 [22,23]. Additionally, based on internal institutional data, approximately 65% of the IP1 students were transitioning to Pharmacy School after completing their pre-pharmacy requirement in 2–3 years of college education.

The overall study findings could serve as the impetus for universities and colleges to assess the level of awareness of the students’ health risk and implement interventions as part of wellness programs. Universities and colleges are in unique positions in the early detection and prevention of risk factors to chronic health problems. This unique role of colleges and universities is underscored by Yahia and colleagues [13].

## 4. Conclusions

The target population’s level of awareness of knowing their health-related numbers was very low. Two or more years of college education and/or choice of a health professional career made some difference in level of awareness. Further research is required to understand the low level of awareness of critical health indicators among the target population.

## Figures and Tables

**Table 1:** Baseline characteristics of students.

Characteristics	Freshmen (*n* = 133)	Pharmacy IP1 (*n* = 90)	Total (*n* = 223)
***Gender (%)***			
Male	12.8	30.0	19.7
Female	87.2	70.0	80.3
***Age in years (%)***			
<18 years	1.5	0.0	1.5
18–24 years	98.5	28.9	70.4
25–34 years	0.0	53.3	21.5
35–44 years	0.0	8.9	3.6
45–54 years	0.0	8.9	3.6
***Race/Ethnicity (%)***			
African American	91.7	37.8	70.0
White	0.0	16.7	6.7
Asian	3.8	43.3	19.7
American Indian	0.8	0.0	0.5
Hispanic/Latino	0.8	0.0	0.5
Other	3.0	2.2	2.7
***Health-Related Numbers***			
Knowledge of total cholesterol level, yes (%)	5.3	10.0	7.2
Knowledge of blood pressure, yes (%)	27.8	45.6	35.0
Knowledge of blood glucose, yes (%)	7.5	8.9	8.1
Knowledge of body mass index (BMI), yes (%)	40.9	43.3	41.9
% with all 4 health indicators in normal range	17.4	31.1	23.0
***Correct response to a reference range for:***			
Total cholesterol for a healthy adult	27.8	30.0	28.7
Blood pressure for a healthy adult	62.9	74.4	67.6
Blood Glucose for a healthy adult	26.3	23.3	25.1
Body Mass Index for a healthy adult	23.1	38.9	29.6
***Health Behaviors***			
Number of days in the past 7 days that the participant engaged in physical activity or exercise for at least 30 min each day (M ± S)	2.28 ± 2.14	2.66 ± 1.68	2.43 ± 1.97
Participant maintains a healthy diet, yes (%)	36.1	64.4	52.5

Note: IP1: Incoming Professional Year 1; M: Sample mean; S: Sample standard deviation.

**Table 2:** Comparative analysis of key outcome measures of knowing your numbers.

Measure	Freshmen	Pharmacy IP1	*p*-Value
***Health-Related Numbers***			
Knowledge of total cholesterol level, yes (%)	5.3	10.0	0.280
Knowledge of blood pressure, yes (%)	27.8	45.6	0.010
Knowledge of blood glucose, yes (%)	7.5	8.9	0.906
Knowledge of body mass index (BMI), yes (%)	40.9	43.3	0.825
***Correct response to a reference range for a healthy adult:***			
Total cholesterol	27.8	30.0	0.334
Blood pressure	62.9	74.4	0.049
Blood Glucose	26.3	23.3	0.456
Body Mass Index	23.1	38.9	0.044
***Composite Measures***			
Knowledge Score of Participant’s Numbers	0.81 ± 0.95	1.08 ± 1.08	0.053
Knowledge Score of Normal Reference Values	1.40 ± 0.10	1.67 ± 0.11	0.082
Physical Activity for at least 30 min each day	2.28 ± 2.14	2.66 ± 1.68	0.147
Participant maintains a healthy diet, yes (%)	36.1	64.4	<0.0001
Number of days in the past 7 days that the participant engaged in physical activity or exercise for at least 30 min each day (M ± S)	2.28 ± 2.14	2.66 ± 1.68	0.147

Note: M: Sample mean; S: Sample standard deviation.

**Table 3:** Association between knowing one’s numbers and knowledge of reference values of healthy adults.

	**Knowledge of Total Cholesterol Reference Values**		**Knowledge of Blood Pressure Reference Values**	
**Variables**	**Model I OR (95% CI)**	**Model II OR (95% CI)**	**Model I OR (95% CI)**	**Model II OR (95% CI)**
Gender	-	0.48 (0.22, 1.03)	-	0.72 (0.35, 1.50)
Age	-	1.67 (0.94, 2.98)	-	2.28 (1.02, 5.07)
Educational Attainment	-	0.75 (0.39, 1.47)	-	1.55 (0.77, 3.11)
Race/Ethnicity	-	0.89 (0.65, 1.22)	-	0.82 (0.60, 1.11)
Study Group (Ref: Freshmen)	1.04 (0.57, 1.90)	0.24 (0.05, 1.15)	1.38 (0.74, 2.58)	0.42 (0.10, 1.71)
Knowledge of Numbers (Ref: No)	3.53 (1.25, 9.98)	2.84 (0.96, 8.36)	5.67 (2.61, 12.29)	6.07 (2.69, 13.66)
	**Knowledge of Blood Glucose Reference Values**		**Knowledge of Body Mass Index Reference Values**	
**Variables**	**Model I OR (95% CI)**	**Model II OR (95% CI)**	**Model I OR (95% CI)**	**Model II OR (95% CI)**
Gender	-	0.94 (0.43, 2.04)	-	0.73 (0.35, 1.54)
Age	-	1.65 (0.92, 2.97)	-	1.27 (0.74, 2.18)
Educational Attainment	-	0.69 (0.34, 1.37)	-	1.20 (0.60, 2.40)
Race/Ethnicity	-	0.94 (0.68, 1.29)	-	0.98 (0.72, 1.35)
Study Group (Ref: Freshmen)	1.20 (0.64, 2.27)	0.57 (0.12, 2.68)	2.16 (1.17, 4.01)	0.98 (0.23, 4.13)
Knowledge of Numbers (Ref: No)	3.40 (1.27, 9.07)	3.05 (1.11, 8.33)	4.06 (2.19, 7.53)	4.16 (2.22, 7.79)

Note: Ref: Reference group; Model I: Study Group and Knowledge of Numbers; Model II = Model I plus Gender, Age, Education Attainment and Race/Ethnicity. OR: odds ratio; CI: confidence interval.

## References

[1] Munoz L.R., Etnyre A., Adams M., Herbers S., Witte A., Horlen C., Baynton S., Estrada R., Jones M.E. (2010). Awareness of Heart Disease Among Female College Students. J. Women Health.

[2] Holland C., Carthron D.L., Duren-Winfield V., Lawrence W. (2014). An experiential cardiovascular health education program for African American college students. ABNF J..

[3] Kruger L., Roeder L., Brubaker K. (2014). Health Assessment Data Collection as Part of a College Wellness Course. Phys. Educ..

[4] Reese S. (2009). Management of heart patients involves all stakeholders. Manag. Healthc. Exec..

[5] American Heart Association (2013). Facts: An Ounce of Prevention: The Value of Prevention. http://www.heart.org/idc/groups/heart-public/@wcm/@adv/documents/downloadable/ucm_461177.pdf.

[6] Winham D.M., Jones K.M. (2011). Knowledge of young African American adults about heart disease: A cross-sectional survey. BioMed Cent. Public Health.

[7] Keating X.D., Guan J., Pinero J.C., Bridges D.M. (2005). A meta-analysis of college students, physical activity behaviors. J. Am. Coll. Health.

[8] Collins K., Dantico M., Shearer N., Mossman K. (2004). Heart Disease Awareness among College Students. J. Community Health.

[9] American Heart Association (2017). The Power of Knowing Your Number. http://www.heart.org/HEARTORG/Conditions/Cholesterol/CholesterolToolsResources/The-Power-of-Knowing-Your-Numbers_UCM_462169_Article.jsp#.WKSp4DjJiYE.

[10] (2017). World Heart Federation. http://www.world-heart-federation.org/what-we-do/awareness/women-and-cvd/facts-tips/know-your-risks-know-your-numbers/.

[11] Becker B.M., Bromme R., Jucks R. (2008). College students’ knowledge of concepts related to the metabolic syndrome. Psychol. Health Med..

[12] Spencer L. (2002). Results of a Heart Disease Risk-Factor Screening among Traditional College Students. J. Am. Coll. Health.

[13] Yahia N., Brown C., Rapley M., Chung M. (2014). Assessment of college students’ awareness and knowledge about conditions relevant to metabolic syndrome. Diabetol. Metab. Syndr..

[14] DeBate R.D., Topping M., Sargent R.G. (2001). Racial and Gender Differences in Weight Status and Dietary Practices among College Students. Adolescence.

[15] Ferrara C. (2009). The College Experience: Physical Activity, Nutrition, and Implications for Intervention and Future Research. J. Exerc. Physiol..

[16] Huang T.T.K., Harris K.J., Lee R.E., Nazir N., Born W., Kaur H. (2003). Assessing overweight, obesity, diet and physical activity in college students. J. Am. Coll. Health.

[17] Racette S.B., Deusinger S.S., Strube M.J., Highstein G.R., Deusinger R.H. (2005). Weight changes, exercise, and dietary patterns during freshman and sophomore years of college. J. Am. Coll. Health.

[18] Pinto B.M. (1995). A stages of change approach to understanding college students’ physical activity. J. Am. Coll. Health.

[19] Suminski R.R., Petosa R., Utter A.C., Zhang J.J. (2002). Physical activity among ethnically diverse college students. J. Am. Coll. Health.

[20] Vittinghoff E., McCulloch C.E. (2007). Relaxing the Rule of Ten Events per Variable in Logistic and Cox Regression. Am. J. Epidemiol..

[21] Nesbitt S., Palmomarez R.E. (2016). Review: Increasing Awareness and Education on Health Disparities for Health Care Providers. Ethn. Dis..

[22] Yusuf A.A., Schommer J.C., Mott D.A., Doucette W.R., Gaither C.A., Kreling D.H. (2011). Association between Student Loan Debt on Graduation, Demographic Characteristics and Initial Choice of Practice Setting of Pharmacists. Innov. Pharm..

[23] Cain J., Campbell T., Congdon H.B., Hancock K., Kaun M., Lockman P.R., Evans R.L. (2014). Pharmacy Student Debt and Return on Investment of a Pharmacy Education. Am. J. Pharm. Educ..

